# Brown Adipose Tissue in the Buccal Fat Pad during Infancy

**DOI:** 10.1371/journal.pone.0089533

**Published:** 2014-02-21

**Authors:** Skorn Ponrartana, Shilpa Patil, Patricia C. Aggabao, Zdena Pavlova, Sherin U. Devaskar, Vicente Gilsanz

**Affiliations:** 1 Department of Radiology, Children's Hospital Los Angeles, Los Angeles, California, United States of America; 2 Department of Pediatrics, Children's Hospital Los Angeles, Los Angeles, California, United States of America; 3 Department of Pathology, Children's Hospital Los Angeles, Los Angeles, California, United States of America; 4 Department of Pediatrics, David Geffen School of Medicine, University of California Los Angeles, Los Angeles, California, United States of America; Albert Einstein College of Medicine, United States of America

## Abstract

**Background:**

The buccal fat pad (BFP) is an encapsulated mass of adipose tissue thought to enhance the sucking capabilities of the masticatory muscles during infancy. To date, no conclusive evidence has been provided as to the composition of the BFP in early postnatal life.

**Objective:**

The purpose of this study was to examine whether the BFP of neonates and infants is primarily composed of white adipose tissue (WAT) or brown adipose tissue (BAT).

**Materials and Methods:**

The percentage of fat in the BFP in 32 full-term infants (16 boys and 16 girls), aged one day to 10.6 months, was measured using magnetic resonance imaging (MRI) determinations of fat fraction.

**Results:**

BFP fat fraction increased with age (r = 0.67; P<.0001) and neonates had significantly lower values when compared to older infants; 72.6±9.6 vs. 91.8±2.4, P<.0001. Multiple regression analysis indicated that the age-dependent relationship persisted after accounting for gender, gestational age, and weight percentile (P = .001). Two subjects (aged one and six days) depicted a change in the MRI characteristics of the BFP from primarily BAT to WAT at follow-up examinations two to six weeks later, respectively. Histological post-mortem studies of a 3 day and 1.1 month old revealed predominantly BAT and WAT in the BFP, respectively.

**Conclusion:**

The BFP is primarily composed of BAT during the first weeks of life, but of WAT thereafter. Studies are needed to investigate the contributions of BAT in the BFP to infant feeding and how it is altered by postnatal nutrition.

## Introduction

Adipose tissue is comprised of a mixture of white and brown adipocytes in newborns. While white adipose tissue (WAT) stores energy, brown adipose tissue (BAT) is specialized in dissipating energy in the form of heat to ensure effective adaptation to the colder extrauterine environment [1]. Compared to white adipocytes, brown adipocytes are smaller, highly vascularized, and characterized by an abundance of mitochondria expressing uncoupling protein-1 (UCP1) and multilocular droplets containing small amounts of fat [1]. Based on these cytological differences in vascularization and lipid content, innovative magnetic resonance imaging (MRI) techniques have been developed to provide reliable and accurate measures of both WAT and BAT [2], [3], [4], even in infants without the need for sedation [5].

The buccal fat pad (BFP) (also known as the Bichat or masticatory fat pad) is an encapsulated mass of adipose tissue located between the masticatory muscles. Its perceived function in fetuses and infants is for sucking and enhancing the capabilities of the buccinators and other masticatory muscles [6], [7]. Early studies have suggested that the difficulty in sucking experienced by some premature and poorly developed infants may be in part due to the incomplete development of the BFP [8]. One previous study reported that adipocytes in the masticatory fat pad, alike brown adipocytes, are smaller and contain more numerous fat vacuoles than those in the subcutaneous depot in human fetuses [9].

To date, no conclusive evidence has been provided as to the composition of the BFP in early postnatal life, and neither the histological structure nor the composition of the BFP has been shown to be different than that of WAT in children and adults [10], [11]. The purpose of this study was to examine whether the BFP of neonates and infants is primarily composed of white or brown adipose tissue.

## Materials and Methods

Ethics Statement: The institutional review board for clinical investigations at Children's Hospital Los Angeles approved the post-mortem and clinical studies, which were compliant with the Health Insurance Portability and Accountability Act, and written informed consent was obtained from the parent/legal guardian(s) of all subjects.

The study population comprises 32 full-term infants (16 male and 16 female; aged one day to 10.6 months), who were born to mothers without a history of diabetes or gestational diabetes mellitus (GDM) and who required MRI studies of the head and/or neck as workup for conditions not known to be associated with changes in body composition. Two infants (1 male and 1 female; aged one and six days old) had follow-up examinations two to six weeks later. For the two postmortem studies, the body was imaged within 12 hours of death and was not frozen. Biopsies of the BFP were also taken, and extracted samples were fixed in formalin, embedded in paraffin, and stained with hematoxylin and eosin for visual analysis under a microscope. Age, height, and weight measures were obtained at the time of each MRI examination. Weight percentiles were computed for the study subjects according to the World Health Organization growth charts. The mother's age, parity, and diabetes and GDM diagnoses were obtained from medical records.

All MRI exams were performed on a 3T whole-body human platform (Achieva, R3.2, Philips Healthcare, Cleveland, Ohio) using a chemical-shift water-fat multi-echo pulse sequence (mDIXON) [12], [13]. Data reconstruction yields co-registered and T2*-corrected fat, water, in-phase, and out-of-phase image series and a quantitative fat fraction (FF) map that accurately reflects the underlying proton density ratios between fat and the sum of fat and water from a range of 0–100% [13], [14]. Previous imaging and histological studies indicate that high FF values greater than 90% are indicative of triglyceride-rich WAT, while FF values of less than 85% are characteristic of predominantly BAT [5]. Image analysis was performed with OsiriX (Pixmeo, Geneva, Switzerland) to compute FF values within the BFP. Attention was given to exclude major blood vessels, bone, and adjacent muscles during segmentation. FF measurements are reflective of the relative tissue density and amount of BAT within the fat pad.

Descriptive and linear regression analyses were performed with Statview software (version 5.0.1; SAS Institute, Cary, NC). In most models, the FF was used as the outcome variable, and age, gender, and anthropometric measures were used as possible independent variables. To exclude the possibility of multi-colinearity, the goodness of fit for the regression models was evaluated using the post-estimation procedures of Stata (StataCorp, College Station, TX). All values are expressed as mean ± SD.

## Results

There were no significant differences in the age or weight and height percentile between the 16 male and 16 female infants; there were also no gender differences in their gestational age or birth weight (Table 1). Regardless of gender, the MRI characteristics of BAT changed with age. While the BFP in neonates was primarily composed of BAT as indicated by low FF, infants older than 1 month had significantly higher FF values characteristic of WAT; 72.6±9.6 vs. 91.8±2.4 respectively, P<.0001.

**Table 1:** Age, anthropometric characteristics, gestational age, and birth weight of 32 infants.

	ALL			Males			Females			P-value
	n = 32			n = 16			n = 16			
Age (months)	3.2	±	3.7	2.6	±	3.8	3.9	±	3.6	0.31
Weight (kg)	5.0	±	2.3	4.6	±	2.2	5.5	±	2.3	0.29
Weight Percentile (%)	29.4	±	30.1	28.2	±	34.6	30.6	±	25.9	0.82
Height (cm)	54.5	±	8.0	55.0	±	8.3	53.8	±	7.7	0.72
Height Percentile (%)	32.5	±	31.5	32.4	±	30.5	32.5	±	34.7	0.99
Gestational Age (weeks)	38.8	±	1.0	38.7	±	0.8	38.8	±	1.1	0.71
Birth Weight (kg)	3.1	±	0.6	3.1	±	0.6	3.1	±	0.5	0.98

**Figure 1:**
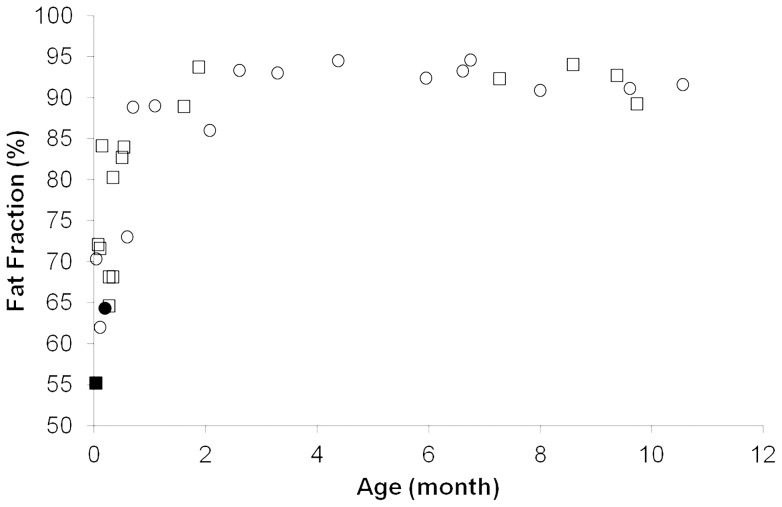
Relationship between fat fraction in the BFP and age. While the mean FF of the BFP increased significantly in neonates from 1(r = 0.64; P = .011), there were no significant differences in FF values among infants older than 1 month of age (r = 0.21; P = .411). The circular and triangular symbols represent male and female infants, respectively, and the solid symbols represent the baseline values for the two longitudinal studies.

There were moderate positive correlations between FF and age (Figure 1); this was true when subjects were analyzed together (r = 0.67, P<.0001) or when males and females were analyzed separately (r's = 0.66 and 0.65, respectively; both P's≤.0064). While the mean FF of the BFP increased linearly in neonates aged 1 day to 1 month (r = 0.64; P = .011), this association was not present in infants older than 1 month of age (r = 0.21; P = .411). Multiple linear regression analyses indicated that the association between FF measures at the BFP and age was independent of gender, gestational age, or weight percentile when all subjects were analyzed together (Table 2).

**Table 2:** Multiple linear regression analysis for the prediction of FF measures in the BFP.

ALL (n = 32)	β	SE	P-value	R^2^
Fat Fraction (%)				
Gender	0.10	3.28	0.50	0.484
Gestational Age (weeks)	0.14	1.71	0.32	
Age (months)	0.70	0.49	<0.0001	
Weight Percentile (%)	−0.09	0.06	0.55	

**Figure 2:**
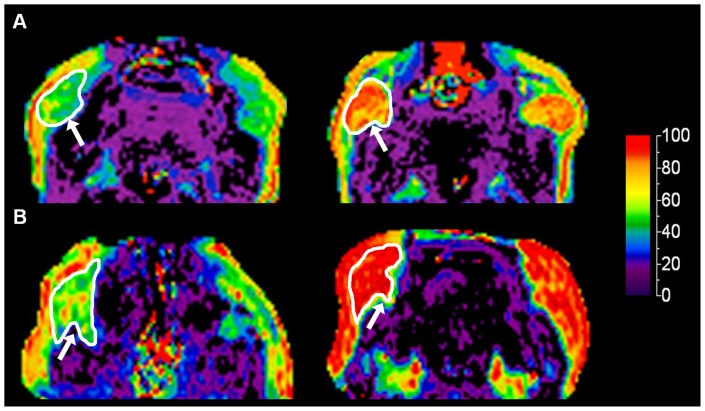
Changes in the composition of the BFP during infancy. Dixon MRI examinations of the BFP (arrow) in a 1 day old male infant (A) and a 6 day old female infant (B) showing increases in FF values from 55 to 84% and 64 to 94% two to six weeks later, respectively.

Figure 2 shows a change in the MRI characteristics of the BFP of two subjects (aged one day and six days) from primarily BAT to WAT at follow-up examinations two to six weeks later; FF values increased from 55.2 to 84.0% and 64.3 to 93.7%, respectively.

**Figure 3:**
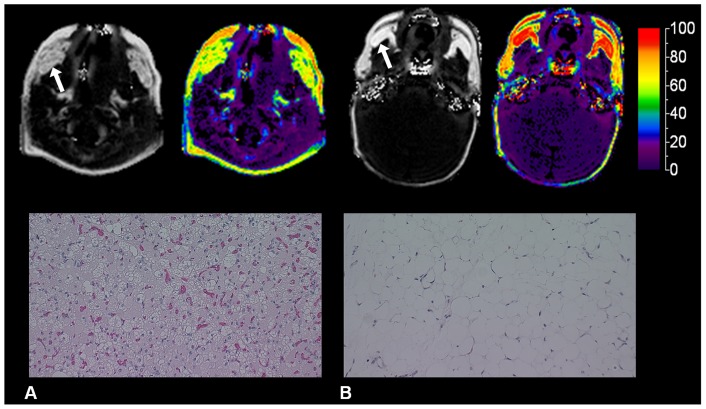
Post-mortem MRI and histological examinations of the BFP. MRI and histological examinations of the BFP (arrow) in post-mortem studies of a 3 day old infant depicting low FF values (green) and small adipocytes, polygonal in shape, with multiple intracellular lipid droplets, characteristic of BAT (A) and a 1.1 month old infant depicting high FF values (red) and large white adipocytes, circular in shape, with single intracellular vacuoles of lipid droplets, characteristic of WAT (B).

MRI and histological post-mortem studies of a three day old infant depicted predominantly BAT, while those in a 1.1 month old infant showed predominantly WAT in the BFP (Figure 3).

## Discussion

The results of the current study indicate that the composition of the buccal fat pad, an important contributor to the prominence of the cheek and to the sucking mechanism of newborns, changes during infancy. While this fat depot predominantly has MRI and histological characteristics of BAT during the first weeks of postnatal life, it has signatures of WAT thereafter. This age-dependency in adipose tissue composition of the BFP was present in both males and females irrespective of gestational age or weight percentile.

The BFP acts as a gliding pad when adjacent masticatory and mimetic muscles contract [15]. Although the physiological relevance of BAT in the BFP of neonates is yet to be clearly defined, brown adipocytes possess a unique uncoupling protein, UCP1, that is responsible for the rapid generation of large amounts of heat. The possibility exists that BAT in the BFP could help warm masticatory muscles and prepare them to activate the energy systems required for sucking. Support for this hypothesis comes from animal studies showing that *Gnasxl* mutant mice had impaired suckling activity coupled with a reduction of BAT [16]. This notion is also consistent with clinical observations showing significant improvements in sucking efficiency during the first month of life concurrent with, as the results of the current study indicate, the presence of BAT in the BFP.

Regardless of the potential mechanism by which brown fat in the BFP enhances the capabilities of the masticatory muscles, available data suggests a close link between BAT and muscle [17]. Classical brown adipocytes derive from myoblastic-like *myf-5* positive precursors, and share many features with skeletal muscle cells, such as an abundance of mitochondria, energy expenditure via oxidative phosphorylation, and sympathetically mediated adaptive thermogenesis [18], [19], [20]. Moreover, brown adipocytes have been identified in skeletal muscle, and their abundance in different strains of mice correlates with differences in energy expenditure [21].

Several factors, including the histology of adipose tissue and the MRI technique used to measure BAT, should be taken into account before interpreting our results. While WAT is characterized by large adipocytes that contain a large unilocular lipid droplet and limited cytoplasm, BAT contains smaller adipocytes with multiple lipid droplets, an abundance of mitochondria, and dense vascularity. These intrinsic morphological differences between BAT and WAT give rise to unique signatures that can reliably be detected and quantified by chemical-shift water-fat MRI [5], [13], [14]. With these techniques, a high FF is indicative of triglyceride-rich WAT, while lower FF values denote greater water content and the presence of brown adipocytes. Our imaging and postmortem findings are consistent with previous histological studies showing that the adipocytes of the BFP are characteristic of WAT in children and adults, but those of prenatal infants (aged 14 to 42 gestational weeks) are smaller and more numerous per unit area when compared to adipocytes in WAT [9].

This study has several notable limitations. It is based on post-mortem studies and the analysis of a heterogeneous group of patients requiring brain examinations. However, in order to minimize the confounding effect of disease, we selected infants with conditions not known to affect body composition. Additionally, we did not control for many important determinants of fetal growth, such as maternal weight and metabolic status, beyond excluding infants of diabetic mothers. We also did not account for the influence of feeding practice even though, when compared to breastfeeding, formula feeding requires less muscle activity and may be associated with altered body composition. Many adipokines in breast milk, such as leptin, adiponectin, and ghrelin have been found to be associated with UCP1 expression in BAT [22], [23], [24], [25], and their concentrations correlate with infant body weight [26].

## Conclusion

This study provides evidence for the temporary presence of BAT in the BFP in early postnatal life. A key question that remains is whether BAT transdifferentiates into WAT in the BFP or WAT simply fills an empty space and replaces BAT over time. Studies are also needed to better determine the degree to which BAT in the BFP is linked to muscle function and infant nutrition, and to establish the significance of this adipose tissue in preterm and low birth weight infants known to lack the coordination between suckling, swallowing, and breathing.
